# Structure before Meaning: Sentence Processing, Plausibility, and Subcategorization

**DOI:** 10.1371/journal.pone.0076326

**Published:** 2013-10-07

**Authors:** Johannes Kizach, Anne Mette Nyvad, Ken Ramshøj Christensen

**Affiliations:** Department of Aesthetics and Communication, Aarhus University, Aarhus, Denmark; University of Leicester, United Kingdom

## Abstract

Natural language processing is a fast and automatized process. A crucial part of this process is parsing, the online incremental construction of a syntactic structure. The aim of this study was to test whether a *wh*-filler extracted from an embedded clause is initially attached as the object of the matrix verb with subsequent reanalysis, and if so, whether the plausibility of such an attachment has an effect on reaction time. Finally, we wanted to examine whether subcategorization plays a role. We used a method called G-Maze to measure response time in a self-paced reading design. The experiments confirmed that there is early attachment of fillers to the matrix verb. When this attachment is implausible, the off-line acceptability of the whole sentence is significantly reduced. The on-line results showed that G-Maze was highly suited for this type of experiment. In accordance with our predictions, the results suggest that the parser ignores (or has no access to information about) implausibility and attaches fillers as soon as possible to the matrix verb. However, the results also show that the parser uses the subcategorization frame of the matrix verb. In short, the parser ignores semantic information and allows implausible attachments but adheres to information about which type of object a verb can take, ensuring that the parser does not make impossible attachments. We argue that the evidence supports a syntactic parser informed by syntactic cues, rather than one guided by semantic cues or one that is blind, or completely autonomous.

## Introduction

Parsing refers to the process of assigning syntactic structure to language stimuli during on-line language comprehension in real time. Basically, each incoming word is integrated incrementally into a syntactic representation of a sentence, a parse tree [1–4]. For each word, the human sentence parser must decide how to integrate it into the current structure, and in the case of potential ambiguity, there are multiple theoretically possible options. A key question in the literature on sentence processing is how the parser makes these decisions; that is, which types of information are taken into account during on-line parsing [1,4–10]. Does the parser rely on a few (syntactic) heuristics, or does it employ all available information? A radical answer to this question is provided by Mitchell [11] and van Gompel and Pickering [12] who demonstrate that noun phrases are attached as direct objects of intransitive verbs (but see 13). For a parser to do this, it must rely on some basic syntactic heuristics, such as *late closure*, which states that the parser prefers to attach new words into the phrase currently being processed [1], and it must ignore (or have restricted access to) non-syntactic information and information about subcategorization frames (i.e. transitivity, requirements on the number and types of objects/complements a verb can or must take). At the other extreme, it has been argued that the human parser has access to all types of information, including syntax, semantics and pragmatics, simultaneously when making attachment decisions [3,14].

A classic method of determining which factors affect attachment decisions is the plausibility paradigm, where a filler (a *wh*-phrase, such as *which rule*, occurring at the left edge of the sentence) is associated with a gap at the end of the sentence, but where a possible gap site intervenes [14,15]. In (1a) below, *which language* is the filler, and (✓) indicates a possible and plausible attachment site; the intermediate attachment site is plausible (*which language* could be the object of *learnt*) though the *wh*-phrase is ultimately attached as the object of *speak* at the final attachment site.

(1)
a. [Which language] has the student learnt (✓) to speak (✓)?    (Plausible)
b. [Which drain] has the student learnt (*) to clean (✓)?     (Implausible)

In (1b), however, the extracted *wh*-phrase, *which drain*, is not a plausible object of the matrix verb, *learnt*, indicated with (*); only the final attachment site yields a plausible interpretation, indicated with (✓).

Using an incremental method of stimulus presentation (e.g. self-paced reading), it is possible to measure localized increases in response time (RT) due to increases in processing cost. If the parser avoids intermediate attachment when the filler is an implausible object, as in (1b), then the prediction is that there would be no anomaly effect giving rise to increased RT at the matrix verb *learn*. On the other hand, if the parser does attach the implausible filler at the intermediate position, an increase in RT at the matrix verb is predicted due to the anomaly effect.

Several studies have investigated whether implausibility blocks intermediate attachment or not. For example, Boland, Tanenhaus, Garnsey, and Carlson [16] found that plausibility affected attachment, whereas Traxler and Pickering [4,17] concluded that it did not. Likewise, for verbs with multiple subcategorization frames, Trueswell, Tanenhaus, and Kello [18] observed an effect of frequency on attachment, whereas Pickering and Traxler [4] found no such effect. In fact, there is currently little agreement as to which factors influence intermediate attachment decisions [13].

Implausible fillers have been found to increase RT at the first (intermediate) attachment site (i.e. when the parser encounters the matrix verb) compared to plausible fillers [10,17]. Arguably, however, this RT effect in itself does not really tell us much about whether attachment takes place or not. If the theoretical assumption is that structural attachment precedes semantic evaluation [19], then clearly the increase in RT could be taken to suggest early attachment. But on the other hand, if the assumption is that various types of information are used simultaneously, then it could be argued that the RT effect is the result of the decision process (i.e. choosing between competing alternatives); other things being equal, it is likely that it takes longer for the parser when the syntactic preference (‘fill the gap’) is in conflict with the semantic preference (‘avoid implausible objects’), compared to when they converge [14].

The crucial question, rather, is whether there is an effect, not at the actual point of early (intermediate) attachment (i.e. at the matrix verb *learnt* in (1) above), but at the subsequent point of disambiguation (i.e. at the infinitive marker *to* in (1)), where reanalysis must occur in case of attachment. This means that a model that assumes that implausibility can hinder attachment predicts a higher RT for the plausible condition at *to* in (1a) because there is attachment and reanalysis. In contrast, a parser that is immune to implausibility and operates solely on syntactic information is predicted to show no difference in RT at *to* since attachment takes place regardless, and there is ultimately reanalysis in both (1a) and (1b). The plausibility paradigm can thus be used to distinguish between what we will refer to as a syntactic parser (i.e. one that relies mostly or solely on syntax in initial attachment) and a semantic parser (one that relies mostly on semantic cues, or at least ranks semantic cues higher than syntactic ones). Note that the question of which sources of information the parser uses to make decisions is largely orthogonal to the question of serial vs. parallel parsing, because both ranked parallel parsers and serial parsers could base their structural preferences on either syntactic or semantic cues [20–23].

A third kind of parser, which we shall refer to as a blind parser, is a fully autonomous syntactic parser that also ignores (or has no access to) subcategorization information [11]. The prediction for the blind parser is the same as for the syntactic parser, namely that early attachment occurs regardless of whether the filler is plausible or not. In order to distinguish between the blind parser and the syntactic parser, we need to look at verbs that show a contrast in their subcategorization frames, as exemplified in (2) below. The matrix verb in (2a), *noticed*, can take either a clausal complement, i.e. a complementizer phrase (CP) ((*that*) *the pig … was missing*), or a noun phrase (or, rather, a determiner phrase, henceforth, DP) object (*the pig*). The matrix verb in (2b), *presumed*, on the other hand, is only compatible with a CP complement (*John presumed the pig is ungrammatical).

(2)
a. John noticed the pig in the pen was missing.    (CP or DP object)
b. John presumed the pig in the pen was missing.    (CP object only)

In (2a), *the pig* is attached as a DP object by all three types of parser, and reanalysis must take place at the disambiguating verb *was*; until this point, *the pig in the pen* is a possible and plausible object. In (2b), however, only the blind parser attaches *the pig* as a direct object. The syntactic and the semantic parsers do not have this option available since information about matrix verb subcategorization is accessible and blocks erroneous early attachment; instead they have to make a more complicated analysis and attach *the pig* as the subject of an embedded clause. Crucially, no reanalysis is needed at *was* for the syntactic and the semantic parsers because there is no attachment at the intermediate potential gap. Hence, with a syntactic or semantic parser, a higher RT is predicted at the disambiguating *was* in (2a) (the CP/DP condition, where there is reanalysis) compared to (2b) (where there is no reanalysis).

In our study we used the plausibility contrast in (1) and the subcategorization contrast in (2) to determine which of the three parsers was most successful in accounting for the data: the syntactic, the semantic or the blind parser. In other words, we aimed to determine which sources of information affect the attachment decisions and, more specifically, whether plausibility and subcategorization requirements affected the parser’s initial attachment decision.

Below we report the results of one off-line acceptability judgment task and two experiments using G-Maze [24]. Based on these results, we shall argue in favor of a syntactic parser.

## Experiment 1

Before we attempted to locate the plausibility effect precisely using self-paced reading, we needed to make sure that the effect really was there in Danish. Experiment 1 was designed to test whether there is an acceptability contrast between fully grammatical sentences with implausible intermediate attachments and sentences without. In an earlier study using acceptability judgments on *wh*-extraction in Danish, we found an effect of plausible vs. implausible intermediate, temporary attachment on overall acceptability such that an implausible intermediate attachment gave rise to lower acceptability of an otherwise fully grammatical sentence [25], see also 26. A similar result is reported by Pickering and Traxler [4] using self-paced reading and eye-tracking. Based on these prior results we expect to find reduced acceptability in the implausible condition, and we assume that the lower acceptability reflects the additional processing load caused by the implausibility of the temporary attachment cf [26].

## Materials and Methods

### Norming of the materials

We constructed 15 sentence-pairs corresponding to (1) above with a *wh*-filler extracted from the gap site in the object position in the embedded clause. We made two versions of each sentence: one where the *wh*-filler is plausible as the object of the matrix verb and one where the *wh*-filler is implausible as the object of the matrix verb. In this way the matrix verb was the same across conditions, but the *wh*-filler and the embedded verb varied:

(3)
a. Hvilket sprog har studenten (✓) lært at tale (✓)?    (Plausible)
*   Which language has student-the learnt to speak*
   ‘Which language has the student learnt to speak?’

b. Hvilket afløb har studenten (*) lært at rense (✓)?    (Implausible)
*   Which drain has student-the learnt to clean*
   ‘Which drain has the student learnt to clean?’

In the self-paced reading experiment (experiment 2) we would need a set of sentence pairs where only the *wh*-filler varied across conditions while all the other words in each pair were kept constant, to avoid word length and frequency effects on RT. We therefore constructed 10 more sentences where only the *wh*-filler differed between the plausible and implausible version:

(4)
a. Hvilken plan har ledelsen afvist (✓) at lægge (✓)?    (Plausible)
*   Which plan has board-the refused to lay*
   ‘Which plan has the board refused to make?’

b. Hvilket gulv har ledelsen afvist (*) at lægge (✓)?    (Implausible)
*   Which floor has board-the refused to lay*
   ‘Which floor has the board refused to lay?’

To make sure that the *wh*-fillers were clearly either plausible or implausible as objects for the matrix verbs, we conducted a norming study. We constructed simple declarative sentences, as in (5), containing the verb-object pairs in the target stimulus set (as in (3)), and these were embedded in a list with 45 filler items.

(5)
a. Studenten lærte et sprog.    (plausible)
*   Student-the learnt a language*
   ‘The student learnt a language.’

b. Studenten lærte et afløb.    (implausible)
*   Student-the learnt a drain*
   ‘The student learnt a drain.’

Using Google Drive (https://drive.google.com) to create an internet survey, we asked participants to rate the likelihood of the sentences on a scale from 1 (*meget usandsynligt*, ‘very unlikely’) to 6 (*meget sandsynligt*, ‘very likely’). Fifty-six people participated in the survey, and we used the results to remove any sentences containing matrix verb-embedded object pairs that were not either clearly likely (mean rating above 4.3) or clearly unlikely (mean rating below 2.7). Three pairs were removed using this procedure. The mean likelihood of the remaining compatible pairs was 5.2, and the mean for the remaining incompatible pairs was 2.1.

In order to make sure that the relation between the embedded verbs and the fillers were in fact unambiguously plausible, we again used Google Drive to create an internet survey where we asked participants to rate the naturalness of simple sentences corresponding to (6) on a scale from 1 (*meget unaturlig*, ‘very unnatural’) to 7 (*meget naturlig*, ‘very natural’).

(6)
a. Ledelsen lagde et gulv    (Plausible)
*   Board-the laid a floor*
   ‘The board laid down a floor.’

b. Ledelsen lagde en plan    (Plausible)
*   Board-the laid a plan*
   ‘The board made a plan.’

Twenty-eight people participated in this pre-experimental norming study, and we used the results to remove any sentences containing embedded verb-object pairs that were not clearly natural (mean above 4). Four sentences (two pairs) were removed using this procedure. The mean naturalness of the remaining compatible pairs was 5.5.

## Materials and Methods

The stimulus set used in the actual experimental acceptability survey consisted of the 40 sentences (20 pairs) remaining after norming plus 24 fillers (12 fillers were judged unacceptable, and 12 were judged to be completely acceptable in previous studies; see examples in (7) below); 64 sentences in total. The task consisted of providing acceptability judgments on a 7-point Likert-scale (1 = *helt uacceptabel*, ‘completely unacceptable’, 7 = *helt acceptable*, ‘completely acceptable’). Using an internet-survey created using Google Drive, we obtained answers from forty-five participants.

(7)
a. Chaufføren røg en flot cykel    (Filler type A: implausible).
*   Driver-the smoked a nice bicycle*
   ‘The driver smoked a nice bicycle’

b. Kvinden elskede mørk chokolade.    (Filler type B: plausible)
*   Woman-the loved dark chocolate*
   ‘The woman loved dark chocolate’

## Results

**Figure 1:**
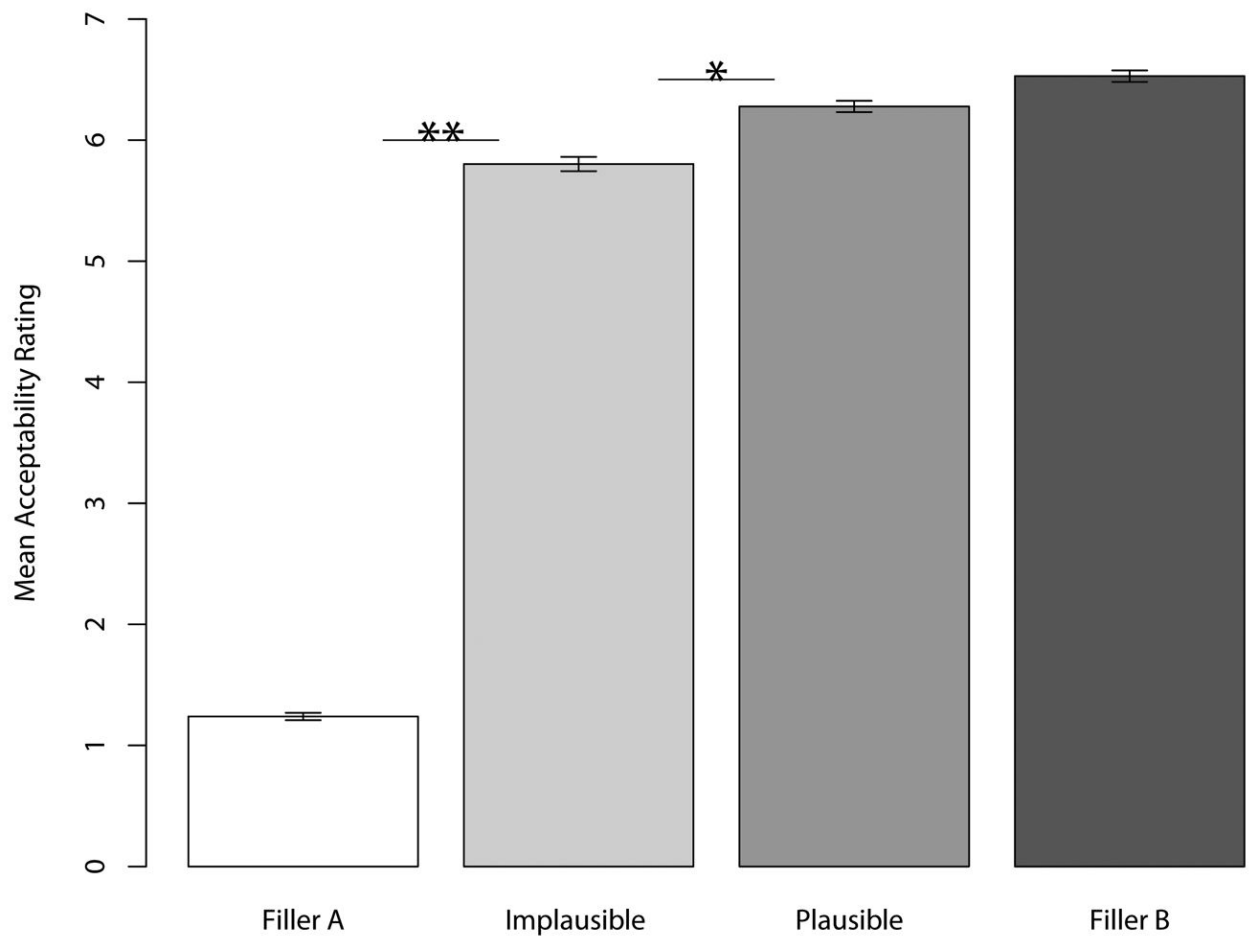
Mean acceptability ratings across sentence types in experiment 2. Error bars ±1 SE. Significant differences: *p<0.05, **p<0.001.

The mean acceptability rating was 6.3 for the plausible condition (corresponding to (4a)), and 5.8 for the implausible condition (as in (4b)). We used a linear mixed-effects regression model to analyze the data with the free software R [27] and the lme4 package for R [28]. The dependent variable was the acceptability rating and the independent variable was the four-level sentence type variable (Filler type A, Filler type B, plausible, and implausible). The model included random intercepts for participants and items. We used contrastive coding to get the pair-wise comparisons of neighboring levels in the sentence type variable [29], and *p*-values were subsequently Holm-corrected for multiple comparisons [30]. The final model is summarized in table 1 (excluding the intercept, which is not crucial here), and the results are illustrated in Figure 1.

**Table 1:** Results of experiment 1.

	**Estimate**	**SE**	***t***	***p***
Filler A vs. Implausible	4.5633	0.2436	18.73	0.0000
Implausible vs. Plausible	0.4756	0.2110	2.25	0.0486
Plausible vs. Filler B	0.2500	0.2436	1.03	0.3049

Summary of the mixed-effects model of acceptability as a function of sentence type. The model included random intercepts for participant (SD = 0.5998) and item (SD = 0.6475) (residual SE = 1.0791).

## Discussion

Experiment 1 confirms that a temporary implausible attachment of a *wh*-filler to a matrix verb has a significant negative effect on the overall acceptability of a sentence. This is consistent with the results reported by Fanselow & Frisch [26] and Christensen et al. [25] where a reduction in acceptability is argued to reflect an increase in processing cost. This causal link between processing cost and acceptability also supports the idea that an early attachment that violates the semantic selectional restrictions of the main verb increases processing cost. Having established that there is a plausibility effect in Danish, the next question was when it occurs in the on-line parsing. Experiment 2 was designed to this end.

### Experiment 2

The plausibility paradigm can, as explained above, be used to separate the semantic parser from the blind and syntactic parsers. The semantic parser predicts that implausible fillers are not attached as objects at the first gap site (immediately after the matrix verb), and consequently no reanalysis is needed at the disambiguating word (the infinitive marker, *to*, in (4a)). The syntactic and blind parsers, on the other hand, predict that fillers are attached as soon as possible, regardless of plausibility, and so reanalysis is required for plausible and implausible fillers alike. Experiment 2 tested whether there was a localized effect at the matrix verb of an implausible temporary attachment of a *wh*-filler extracted from an embedded clause.

## Materials and Methods

We used a subset of the sentences that appeared in experiment 1 (all sentences were normed as described above), namely the 16 sentences (8 pairs) where the plausible and implausible versions of the sentences differed from one another in one respect only: the *wh*-phrase, as in (4) above. In this way we could be certain that any differences in reaction time (RT) could not be attributed to differences in word length or frequency (since all words except the *wh*-phrase were identical in the two conditions). The stimuli were divided into two lists, ensuring that participants saw only one version of each sentence (plausible or implausible). The non-targets (see below) were the same in the two lists, so the plausible and implausible version of each sentence appeared with identical non-targets. The same set of fillers appeared on both lists (14 sentences from an unrelated experiment, and 12 distractor items). Sixty native speakers of Danish participated in this experiment (mean age 23.7, range 17-39). The stimuli were presented in randomized orders (a unique random order for each participant) on a PC running DMDX [31]. Prior to the actual session, a training session was run to familiarize participants with the task. The entire session lasted approximately 6 minutes.

**Figure 2:**
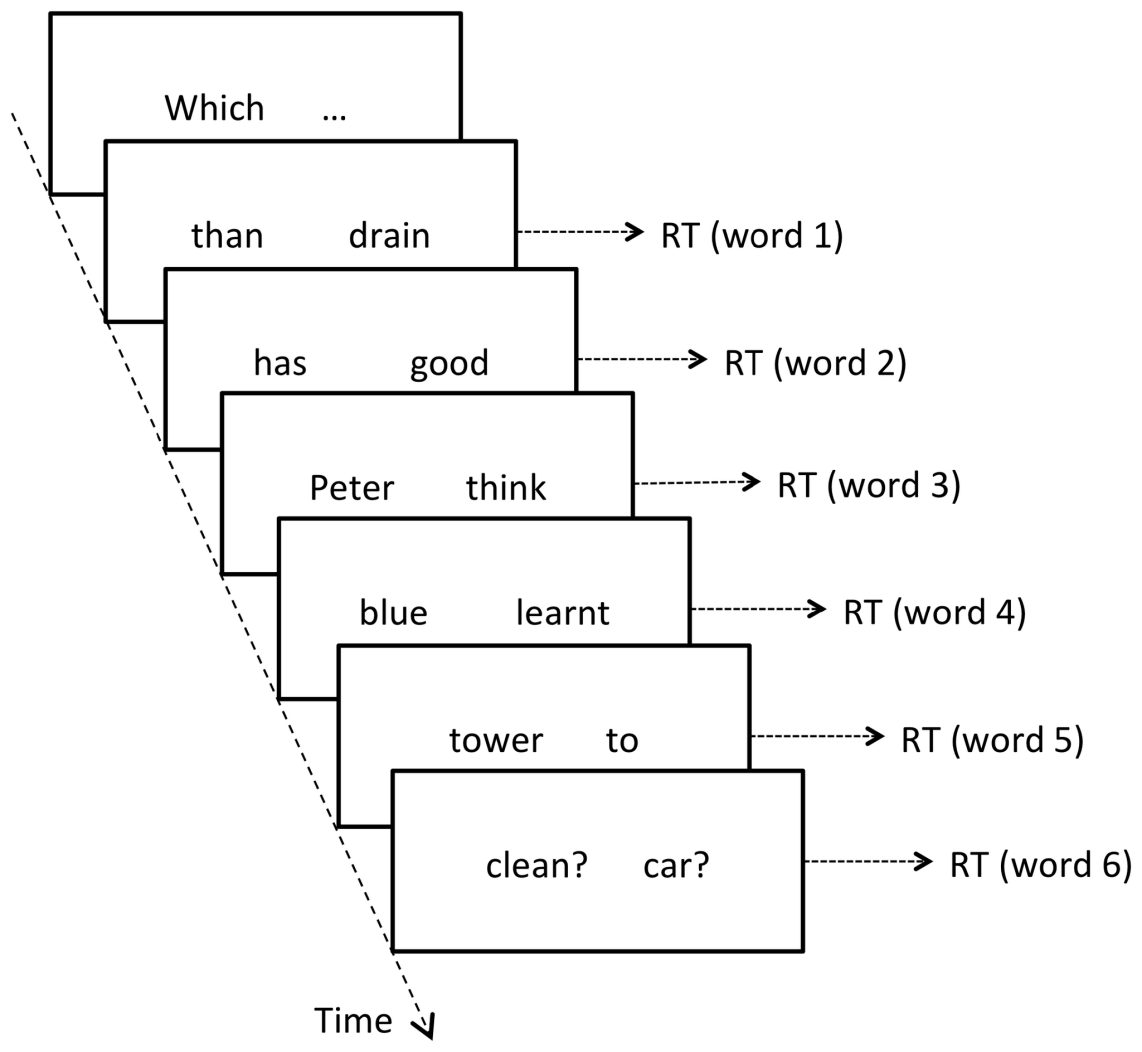
The G-Maze experimental design. Words are presented incrementally in pairs and participants have to select the one that fits the context best. RT is logged for each selection.

The G-Maze method was developed by Forster, Guerrera and Elliot [24] as a low-tech tool to localize processing effects giving rise to increases in RT, and Witzel, Witzel and Forster [32] report results demonstrating that the G-Maze method is comparable to eye tracking and superior to moving window self-paced reading in precision. Sentences are presented one word at the time, and each word is paired with a word that cannot possibly be a continuation of the sentence (a non-target). The task is to choose, using left and right arrow on the keyboard, the target word that continues the sentence in a grammatically possible way (see Figure 2). G-Maze enforces word-by-word incremental processing, and RT is recorded for each word pair presented to the participants, making it possible to localize any RT effects due to processing cost.

Feedback was displayed on the screen immediately after an incorrect choice and at the end of the sentence in the case of only correct choices. After feedback, participants pressed the spacebar to continue the experiment.

## Results

**Figure 3:**
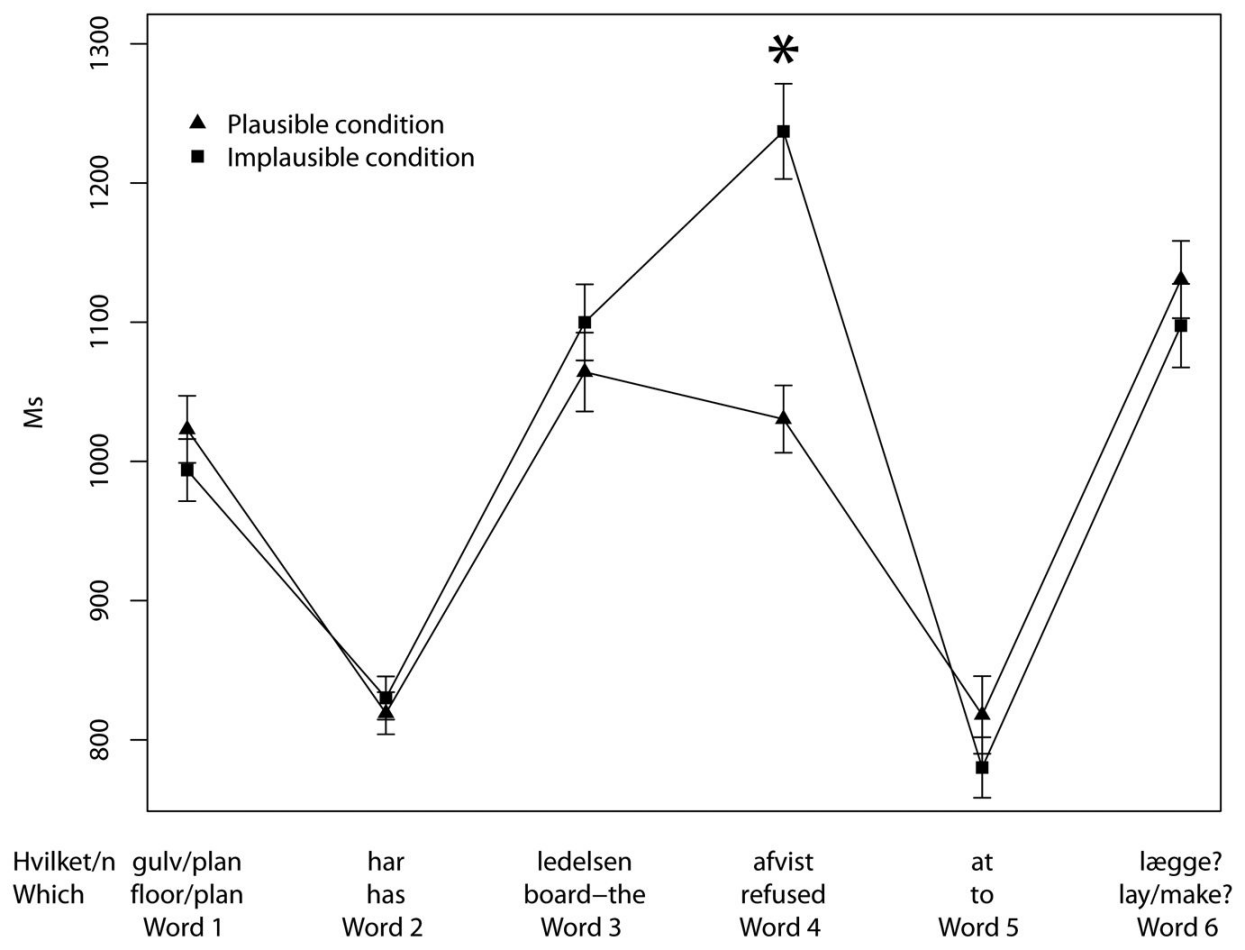
Mean RT, word for word, experiment 2. Error bars ± 1 SE, *p<0.05.

The data was analyzed using a linear mixed-effects regression model (same software as in experiment 1). The dependent variable was RT, and intercepts for participants and items were included as random effects. The fixed effects were added one by one in following order (reflecting relative theoretical importance, from least to most important): sex, age, previous RT, trial, word position (word 1 to 6 in the sentence) and plausibility. Only significant effects were kept in the model. The final model is summarized in table 2 below (leaving out contrasts between the intercept and each word as they are irrelevant in this context). The results are illustrated in Figure 3.

**Table 2:** Results of experiment 2.

	**Estimate**	**SE**	***t***	***p***
Previous RT	0.0001	0.0001	3.58	0.0003
Trial	-0.0006	0.0001	-6.60	0.0000
Word 1: plausible vs. implausible	0.0260	0.0684	0.38	0.7040
Word 2: plausible vs. implausible	-0.0101	0.0661	-0.15	0.8789
Word 3: plausible vs. implausible	-0.0266	0.0661	-0.40	0.6870
Word 4: plausible vs. implausible	-0.1442	0.0662	-2.18	0.0294
Word 5: plausible vs. implausible	0.0580	0.0663	0.87	0.3817
Word 6: plausible vs. implausible	0.0449	0.0663	0.68	0.4986

Summary of the mixed-effects model of RT in experiment 2 as a function of trial, word position and plausibility. The model included random intercepts for participant (SD = 0.1555) and item (SD = 0.1239) (residual SE = 0.2461).

We found a small familiarization or training effect such that participants respond faster later in the experiment (see Trial row in table 2). We also found an effect of Previous RT, meaning that there was a significant correlation between a slow RT and a likewise slow previous RT, and vice versa for fast responses. This effect simply shows that fast responders were consistently fast and slow responders were consistently slow, even when the random intercepts for participants are taken into consideration.

The only significant effect of plausibility was located at the fourth word, the matrix verb (see Figure 3), where participants responded faster in the plausible condition than in the implausible condition (*p*=0.0294). There was no significant difference at the disambiguating word (word 5, *p*=0.3817) or at the embedded verb (word 6, *p*=0.4986).

## Discussion

The result is in accordance with the predictions of both the blind parser and the syntactic parser; there is no difference at the disambiguating word, i.e. word 5 in Figure 3 (nor at the embedded verb, word 6). The lack of a difference at the disambiguating word is expected if reanalysis occurs in both cases, and the result thus supports this assumption.

The effect of plausibility at the matrix verb (word 4 in Figure 3) is consistent with both the view that semantic evaluation occurs after syntactic attachment (as argued in [19]), and with the view that the simultaneous consideration of syntactic and semantic cues prolongs processing in case of conflict [14]. It is, however, as argued in the introduction, the null-effect at the disambiguating word that reveals that attachment occurs in both conditions. The result suggests that plausible and implausible *wh*-fillers alike are attached as soon as possible. Similarly, in a self-paced reading experiment investigating whether plausibility affects attachment in English, Stowe, Tanenhaus and Carlson [10] found no RT difference at the disambiguating word.

The question is to which degree the parser ignores semantic information: Does the parser also attach impossible elements, or does it only attach implausible elements? In other words, is the parser syntactic or is it blind? To answer this, we designed experiment 3.

### Experiment 3

It has been argued that the parser not only ignores plausibility, but also ignores syntactic subcategorization frames: information about which type of object or complement a verb can take [11,12]; for example, the verb *to ride* takes a DP object (*Johnny rides a bike*), *wonder* takes a sentence (i.e. a CP) (*Sarah wondered if Johnny could ride the bike*) whereas *expect* takes either a DP or a sentence, CP or IP (*Jane expected her friends / her friends to/would be late*). Recently, however, Staub [13] has presented evidence to the contrary, i.e. that information about subcategorization is in fact taken into account in online parsing (see 15 for discussion). Because G-Maze measures RT incrementally, word for word, it can be used to test whether or not the parser ignores (or has no access to) subcategorization frames.

The predictions for verbs that can take either a DP or a CP complement vs. verbs that only take a CP complement are clear: If the stimulus is a sentence with an embedded clause beginning with a noun phrase, e.g. *Jane expected her friends would be late*, the syntactic parser predicts an RT difference at the disambiguating word (*would*), such that processing should be slower for sentences with verbs that can take a DP object (reanalysis increases RT). (Here, the semantic parser makes the exact same predictions as the syntactic parser.) The blind parser predicts no difference at the disambiguating word, because attachment occurs even when impossible.

## Materials and Methods

We constructed 10 sentence-pairs, where each pair only differed with regard to the matrix verb. Either the matrix verb subcategorized for a DP or a CP complement, such as *hørte* ‘heard’ in (8a), or it subcategorized for a CP complement only, *tænkte* ‘thought’ in (8b):

(8)
a. Filosoffen hørte forelæsningen om etik vakte begejstring.    (CP/DP)
*   Philosopher-the heard lecture-the about ethics evoked enthusiasm*
   ‘The philosopher heard the lecture on ethics evoked enthusiasm.’

b. Filosoffen tænkte forelæsningen om etik vakte begejstring.    (CP only)
*   Philosopher-the thought lecture-the about ethics evoked enthusiasm*
   ‘The philosopher thought the lecture on ethics evoked enthusiasm.’

In some cases verbs that usually cannot take a DP object, may, however, take a so-called cognate object. For example, you can think clever thoughts, but you cannot think clever people; likewise, you can die a certain death or sleep the sleep of the righteous, but you cannot die a certain fate or sleep a nap. Given that these cases are very limited, and to a large extend idiomatic, the possibility of a cognate object is probably not a factor in online parsing (unless it is clearly facilitated by the context). At any rate, the (im)possibility of a cognate object is substantially different from normal subcategorization for DP objects and is not relevant here.

Thirty-two people participated in the experiment with ages ranging between 21 and 49 (mean age 26.6 years).

## Results

**Figure 4:**
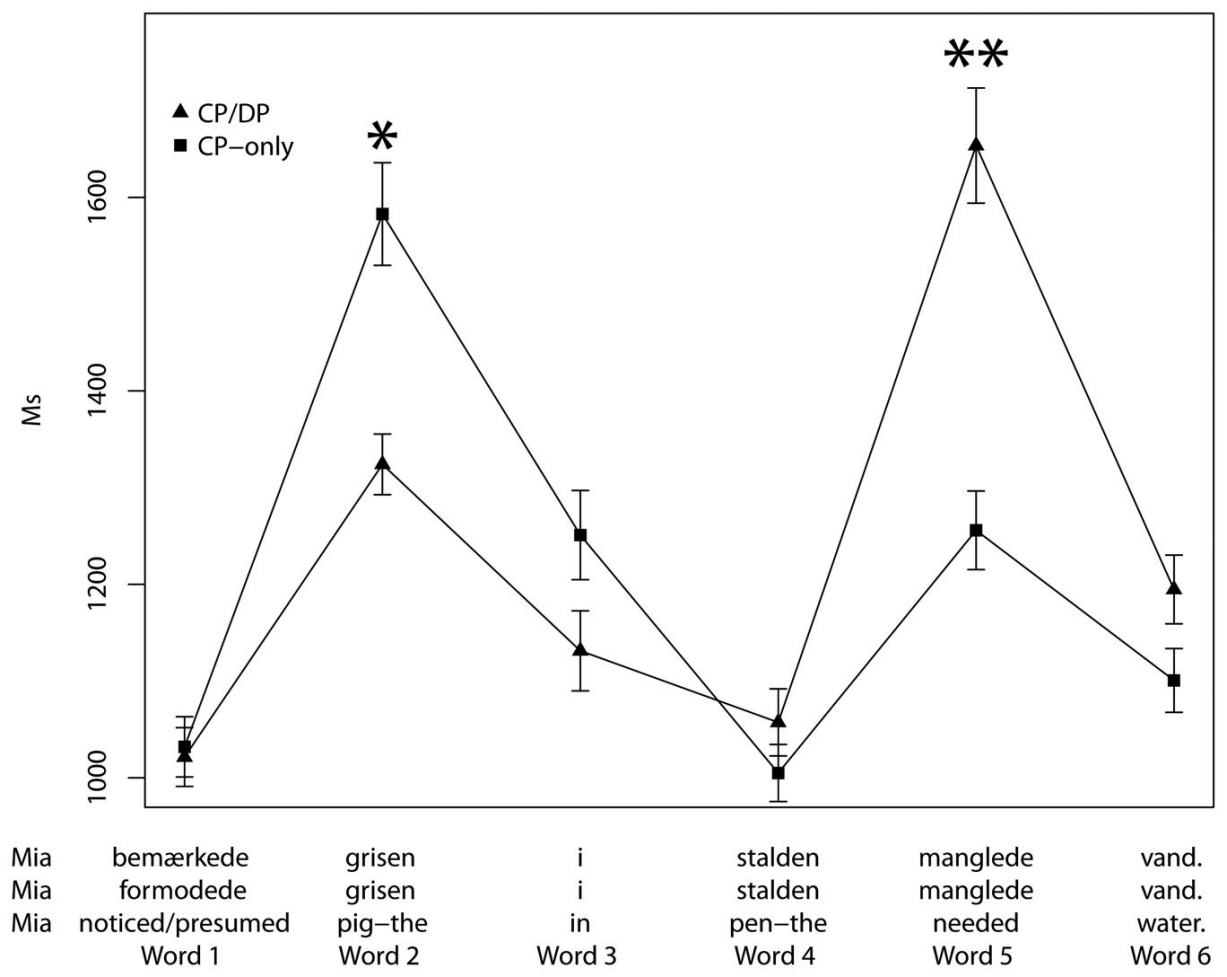
Mean RT, word for word, experiment 3. Error bars ± 1 SE, *p<0.05, **p<0.001.

We fitted a linear mixed-effects regression model to RT using trial, verb type (CP/DP or CP-only) and word position (words 1 to 6) as independent variables (using the same software as in experiment 1). The final model is summarized in Table 3 and the results are illustrated in Figure 4.

**Table 3:** Results of experiment 3.

	**Estimate**	**SE**	***t* value**	***p* value**
Trial	-0.0004	0.0001	-4.68	0.0000
Word 1: CP/DP vs. CP-only	-0.0091	0.0735	-0.12	0.9011
Word 2: CP/DP vs. CP-only	-0.1520	0.0737	-2.06	0.0394
Word 3: CP/DP vs. CP-only	-0.1097	0.0738	-1.49	0.1375
Word 4: CP/DP vs. CP-only	0.0418	0.0739	0.57	0.5717
Word 5: CP/DP vs. CP-only	0.2568	0.0746	3.44	0.0006
Word 6: CP/DP vs. CP-only	0.0856	0.0748	1.14	0.2525

Summary of the mixed-effects model of RT in experiment 4 as a function of trial, word position and verb type (CP/DP vs. CP-only). The model included random intercepts for participant (SD = 0.1494) and item (SD = 0.1502) (residual SE = 0.2663).

Again, we found an effect of trial, suggesting a small, but significant training effect. More interestingly, there was an effect of verb type at both the subject of the embedded clause (word 2 in Figure 4) and at the embedded verb (word 5). At the embedded subject, RT is higher for the CP-only condition (*p*=0.0394), whereas RT is higher at the embedded verb for the CP/DP condition (*p*=0.0006).

## Discussion

The syntactic parser predicts that reanalysis should only be necessary in the CP/DP condition, where the subject of the embedded clause (word 2) is first attached as an object of the matrix verb, and then later (upon reading word 5) reanalyzed to be the subject of the matrix clause. The blind parser, on the other hand, predicts attachment in spite of a potentially conflicting subcategorization frame, and hence, reanalysis in both conditions. The clear slowdown at the embedded verb (word 5, the disambiguating word) in the CP/DP condition is easily explained as reflecting the extra cost of reanalysis. This would mean that incorrect attachment does not take place in the CP-only condition, in accordance with the predictions of the syntactic parser, the one that is informed by syntactic information including subcategorization frames.

**Figure 5:**
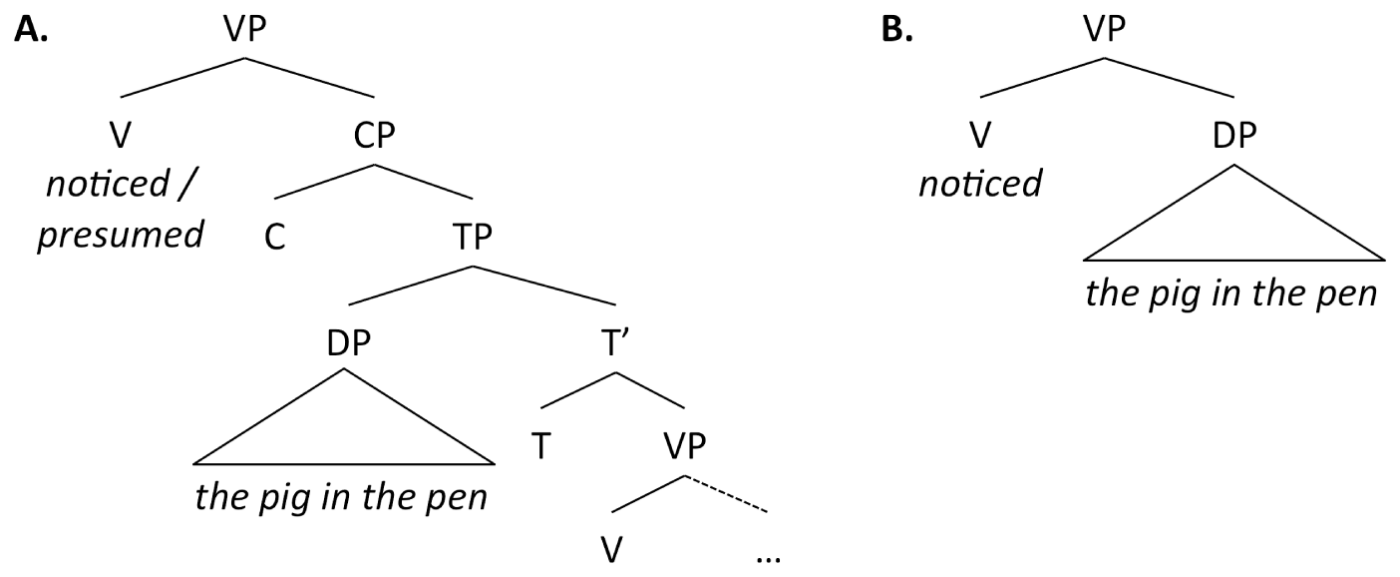
Partial syntactic trees. (A) The structure of a verb phrase headed by a verb with a CP complement, (B) a verb with a DP object. Notice that the verb *notice* appears in both (A) and (B). CP=Complementizer Phrase, TP=Tense Phrase, VP=Verb Phrase, DP=Determiner Phrase. In English, in examples like the ones used here, C can be realized by the optional complementizer *that*, T as a finite auxiliary or tense inflection, and V is where the lexical verb is inserted.

The other significant difference is at the embedded subject (word 2) in the CP-only condition. One could argue that the CP-only condition increases RT due to an anomaly effect from the evaluation of the alternative structures (i.e. ruling out the impossible DP object analysis) [14]; however, we take it to be a reflex of the fact that constructing a clause probably involves more processing than attaching an object, as demonstrated by Staub [13]. (Further support comes from a neuroimaging study by Shetreet, Friedmann, and Hadar [33] that showed increased brain activation for clauses compared to corresponding and matching nominal strings.) The number of syntactic nodes (branchings in the tree structure) has been suggested to be a measure of syntactic complexity [34–37]. Considering the syntactic complexity involved in constructing a CP vs. a DP, it is clear that a CP involves more structure than a DP, see Figure 5. A CP has (at least) an additional three maximal projections (XPs), namely, CP, TP and VP. Constructing a CP is in this respect a much more complex process, and the fact that the RT is higher at word 2 could be a reflection of precisely this.

Hawkins [35] argues that the parser projects (or constructs) a CP if and only if it encounters a word that uniquely requires the presence of a CP-node, such as a complementizer (e.g. *that* or *if*), a *wh*-word (e.g. *what* or *where*) or a nominative pronoun (such as *he* or *she* which must be an embedded subject, in contrast to *him/her* which must be the object). In our stimuli, neither is present (by deliberate design in order to ensure the possibility of ambiguity). The parser has to assume a CP at the second word (word 2 in Figure 4) in the CP-only conditions, despite the lack of a clear, unambiguous input, and this could contribute to the higher RT. Related to this issue is the observation that CP-only verbs are most frequently followed by a complementizer (*at* ‘that’) or a personal pronoun with nominative case (e.g. *han* ‘he’) ( [36], pp. 49-61). To test whether this observation also holds for Danish, we searched for the CP-only verb *håbe* ‘hope’ in the Danish on-line corpus KorpusDK and examined fifty random examples where the verb was used with a CP complement. In 44 cases (88%), the first word in the CP was indeed either a complementizer or a personal pronoun. That is, the parser predicts that a clausal complement is introduced by a complementizer, and when that expectation or preference is not met, there is an error signal and the integration cost goes up with a concurrent increase in RT [38].

Note also that Danish orthography has two alternative comma systems; the ‘grammatical comma’, with obligatory commas before and after embedded clauses (including complement and relative clauses), and the ‘new comma’, where there are only commas after embedded clauses (except parenthetical relative clauses where the initial comma is obligatory). This may potentially affect RT at the embedded subject if (at least some) participants preferred the ‘grammatical comma’ and therefore detected a missing comma, giving rise to an additional error signal [13].

### General discussion

The results presented in this study are consistent with a parser that attaches fillers as soon as possible. As shown in experiment 1, when this early attachment leads to an implausible partial main clause interpretation, the overall acceptability of the full sentence is reduced (Figure 1). The results also support the notion of a syntactic parser that relies on subcategorization frames but ignores plausibility. As illustrated in table 4, the syntactic parser is more successful than its competitors across the three experiments.

**Table 4:** Summary of the successfulness of the three types of parser across experiments.

**Experiment**	**Semantic parser**	**Syntactic parser**	**Blind parser**
1: Plausibility & acceptability	+	+	+
2: Plausibility & RT	–	+	+
3: Subcategorization & RT	+	+	–

A plus (+) indicates that the predictions were borne out in the results, a minus (-) that they were not.

The parser assumed in the classic garden-path model [1,20] and the parser endorsed by Fodor and Inoue [5] are both examples of what we call a syntactic parser. These parsers rely on syntactic cues and choose the syntactically possible analysis that involves the least complexity [35]. This means that when the matrix verb is processed in experiment 2 (see Figure 3), the parser can either attach the *wh*-phrase immediately (ignoring whether the interpretation is plausible or implausible) and not construct any additional syntactic structure, or it can anticipate an embedded clause and construct several new nodes (compare the two trees in Figure 5). The most economical option is to attach the *wh*-phrase as the object, which is predicted to lead to reanalysis in both the plausible and implausible conditions; this prediction is borne out in experiment 2.

In the CP/DP condition in experiment 3, when the DP immediately following the matrix verb is processed, the parser has a choice between analyzing the DP either as the object of the matrix verb or as the subject of an embedded clause (which turns out to be the correct analysis). Clearly, the object analysis involves the fewest new nodes (Figure 5) and is chosen for reasons of computational economy but it results in reanalysis at the embedded verb, cf. Figure 4. In the CP-only condition, on the other hand, the parser has no choice but to construct an embedded clause, and this costly analysis (involving several new nodes) slows the processing down, as observed. The slowdown could also be caused by the fact that a complementizer (or a personal pronoun) is expected in this position, so the appearance of a noun may be unexpected, or by the fact that some participants may have expected a comma after the verb. In any case, the increased RT at the embedded subject in the CP-only condition (word 2 in Figure 4) is not that surprising.

Apart from the theoretical relevance, the results also support the claim that the G-Maze method is a viable alternative to the eye-tracking method [32]. The predicted effects (or null-effects) were found in the exact conditions and at the exact word positions that were expected.

To summarize: The experiments show that the lower acceptability of sentences with an implausible temporary object assignment as compared to sentences with a plausible temporary object assignment is reflected in processing as a lower RT at the matrix verb. There is no difference at the disambiguating word, suggesting that there is reanalysis in both conditions, which means that plausibility does not affect attachment. Subcategorization, on the other hand, does affect attachment, demonstrated by the fact that we find a much slower RT at the disambiguating word in the CP/DP condition, than in the CP only condition.

These results are fully compatible with a syntactic parser informed by syntactic cues and subcategorization frames, not with a semantic parser, nor with a fully autonomous (‘blind’) parser.
